# Characterizing Abdominal Pain and Irritable Bowel Syndrome Among Individuals With Cirrhosis: Results of a Nationwide Survey

**DOI:** 10.1016/j.gastha.2026.100976

**Published:** 2026-04-20

**Authors:** Lisa X. Deng, Christopher V. Almario, Jennifer C. Lai, Brennan M.R. Spiegel, Jessica B. Rubin

**Affiliations:** 1 Department of Medicine, University of California, San Francisco, San Francisco, California; 2 San Francisco Veterans Affairs Health Care System, San Francisco, California; 3 Department of Medicine, Cedars-Sinai Medical Center, Los Angeles, California

Up to 80% of patients with cirrhosis experience at least 1 gastrointestinal (GI) symptom.^1^ Abdominal pain, in particular, affects an estimated 24%–44% of this population, yet remains frequently undertreated.^1^^,^^2^ Emerging evidence suggests shared pathophysiologic mechanisms between irritable bowel syndrome (IBS) and metabolic dysfunction–associated steatotic liver disease, but the prevalence of IBS in cirrhosis has not been established.^3^ Using nationally representative contemporary survey data, we characterized rates and risk factors for abdominal pain and Rome IV-defined IBS among adults with cirrhosis.

We conducted a post hoc analysis of the National GI Survey II, an online survey completed by a representative sample of 88,607 US adults in May–June 2020.^4^, ^5^, ^6^ The study received approval from the Cedars-Sinai Institutional Review Board (Pr056183) and adhered to the Strengthening the Reporting of Observational Studies in Epidemiology guidelines. Aside from age ≥60 years, characteristics of the study cohort were similar to the US population across most sociodemographic strata, including sex, race/ethnicity, and US region, among others; see supplementary material in our prior publication.^5^ Participants self-reported data on demographics, comorbidities (including cirrhosis), and GI symptom severity and frequency, measured using validated instruments: the National Institutes of Health GI Patient-Reported Outcome Measurement Information System (PROMIS) scales and Rome IV IBS questionnaires. We compared GI symptom prevalence in respondents with and without cirrhosis and used multivariable logistic regression to identify predictors of abdominal pain and IBS in this population. Additional methods are detailed in Supplementary Materials.

Among 88,607 survey respondents, 1834 (2%) reported a diagnosis of cirrhosis. Demographic and clinical characteristics of the survey respondents are summarized in Supplementary Table. Abdominal pain was more common in the cirrhosis group (19% vs 17% reported abdominal pain in the last 7 days, *P* = .02). The prevalence of Rome IV IBS was similar among adults with and without cirrhosis (5% vs 6%, *P* = .01). IBS subtype distribution was similar between those with and without cirrhosis: constipation-predominant (4% vs 2%), diarrhea-predominant (3% vs 2%), and mixed type (3% vs 3%). In multivariable models including the full cohort, cirrhosis itself was not an independent predictor of abdominal pain (adjusted odds ratio [aOR]: 0.90; 95% confidence interval [CI]: 0.79–1.02) or IBS (aOR: 0.71; 95% CI: 0.57–0.89).

Respondents with cirrhosis reported higher rates of both upper and lower GI symptoms, including dysphagia (10% vs 5%, *P* < .001), nausea or vomiting (13% vs 8%, *P* < .001), fecal incontinence (7% vs 3%, *P* < .001), pelvic pain (10% vs 4%, *P* < .001), and rectal pain (8% vs 3%, *P* < .001). Those with cirrhosis reported lower rates of heartburn/acid reflux (16% vs 22%, *P* < .001), while rates of abdominal bloating, regurgitation, diarrhea, and constipation were similar between groups.

Individuals with cirrhosis reported higher severity of GI symptoms based on PROMIS percentile scores: abdominal pain (mean abdominal pain PROMIS score 62 vs 59, *P* = .02), dysphagia (65 vs 49, *P* < .001), nausea or vomiting (68 vs 52, *P* < .001), heartburn/acid reflux (71 vs 51, *P* < .001), abdominal bloating (50 vs 36, *P* < .001), constipation (58 vs 46, *P* < .001), fecal incontinence (69 vs 56, *P* < .001), and diarrhea (60 vs 45, *P* < .001) (Figure).

**Figure:**
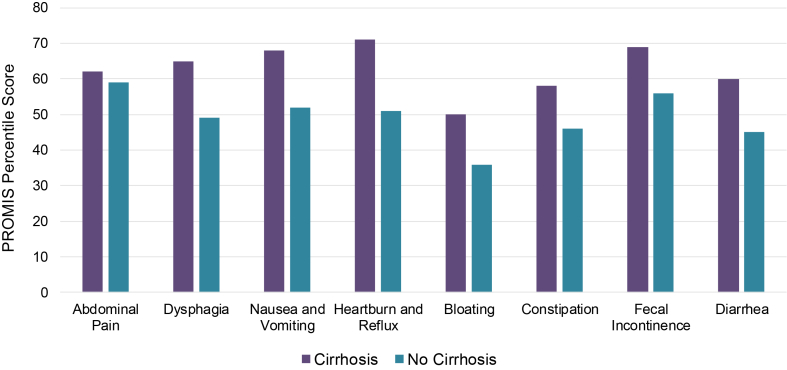
Gastrointestinal symptom severity among adults with and without cirrhosis. All *P* ≤ .02.

In multivariable models limited to the cirrhosis subgroup, factors associated with abdominal pain included being non-Hispanic white (aOR: 1.84; 95% CI: 1.41–2.40), employed or a full-time student (vs unemployed, aOR: 1.68; 95% CI: 1.28–2.22), daily tobacco use (vs some or no use, aOR: 1.39; 95% CI: 1.01–1.92), diabetes (aOR: 1.93; 95% CI: 1.30–2.87), pancreatitis (aOR: 2.65; 95% CI: 1.75–4.01), inflammatory bowel disease (aOR: 1.74; 95% CI: 1.27–2.38), and fibromyalgia (aOR: 1.93; 95% CI: 1.42–2.63) (Table). Likewise, being employed or a full-time student (aOR: 1.77; 95% CI: 1.08–2.90) and comorbid pancreatitis (aOR: 2.34; 95% CI: 1.19–4.63) were also associated with IBS (Table). Predictors of abdominal pain and IBS in the full cohort were similar to those identified in the cirrhosis subgroup (model not shown but comparable to previously published work).^5^

**Table:** Factors Associated With Abdominal Pain^a^ and Rome IV Irritable Bowel Syndrome on Multivariable Models

Variable	Abdominal pain	Rome IV IBS
	aOR (95% CI)	aOR (95% CI)
Age, y		
18–29	1.14 (0.77–1.68)	**0.28** (**0.14–0.55)**
30–49	0.83 (0.58–1.20)	**0.55** (**0.32–0.94)**
>60	Reference	Reference
Gender		
Male	Reference	Reference
Female	1.10 (0.84–1.42)	1.17 (0.73–1.86)
Prefer not to answer	0.61 (0.24–1.52)	1.28 (0.36–4.53)
Race/ethnicity		
Non-Hispanic white	**1.84** (**1.41–2.40)**	1.24 (0.77–2.00)
Non-white	Reference	Reference
Education level		
High school or less	Reference	Reference
Some college	1.32 (0.94–1.87)	1.76 (1.00–3.11)
College graduate	**1.62** (**1.16–2.28)**	1.26 (0.67–2.34)
Graduate degree	1.48 (0.96–2.29)	0.79 (0.31–2.01)
Married	1.17 (0.88–1.56)	1.00 (0.61–1.65)
Employed or full-time student	**1.68** (**1.28–2.22)**	**1.77** (**1.08–2.90)**
Total household income		
$0–$50,000	Reference	Reference
$50,001–$100,000	0.78 (0.57–1.08)	**0.55** (**0.31–0.99)**
$100,001–$200,000	0.68 (0.46–1.01)	0.48 (0.22–1.04)
≥$200,001	1.16 (0.72–1.87)	0.89 (0.38–2.06)
Prefer not to say	0.81 (0.39–1.67)	–
Inflammatory bowel disease	**1.74** (**1.27–2.38)**	–b
Diabetes	**1.93** (**1.30–2.87)**	1.32 (0.76–2.30)
Fibromyalgia	**1.93** (**1.42–2.63)**	0.73 (0.30–1.77)
Pancreatitis	**2.65** (**1.75–4.01)**	**2.34** (**1.19–4.63)**
Alcohol use		
None	Reference	Reference
Some days	0.84 (0.63–1.12)	1.02 (0.61–1.72)
Every day	0.83 (0.50–1.37)	1.10 (0.49–2.49)
Tobacco use		
Not at all	Reference	Reference
Some days	0.86 (0.62–1.19)	0.73 (0.41–1.31)
Every day	**1.39** (**1.01–1.92)**	1.04 (0.60–1.81)

Bolded values indicate statistical significance (P < .05).

a Abdominal pain over the past 7 days.

b Adults with inflammatory bowel disease were excluded from IBS diagnosis.

In this large, nationally representative survey, individuals with cirrhosis reported similar rates of abdominal pain and IBS after adjusting for covariates. Despite similar rates, those with cirrhosis reported higher severity of abdominal pain and other GI symptoms. These findings suggest that the elevated symptom burden in this population may be driven by shared demographic, behavioral, and comorbid factors, rather than cirrhosis or its complications alone. This aligns with prior studies showing that abdominal pain is common in cirrhosis, even in the absence of physiologic causes such as ascites or spontaneous bacterial peritonitis.^2^

Notably, we identified several potentially modifiable risk factors, including type 2 diabetes and tobacco use, to be associated with abdominal pain among individuals with cirrhosis. Diabetes is a known risk factor for metabolic dysfunction–associated steatotic liver disease as well as abdominal pain. Poor glycemic control has been associated with higher prevalence GI symptoms among patients with diabetes.^7^ As for tobacco use, our observations are consistent with a systematic review that found that smoking is a risk factor for abdominal pain.^8^ Our findings have important implications for GI symptom management in cirrhosis. Addressing shared risk factors—such as through glycemic control or tobacco cessation—may offer therapeutic benefit, alongside pharmacologic and nonpharmacologic strategies.^9^

Our study has notable limitations. Comorbidities, including cirrhosis, were self-reported; however, prior work has shown that self-report of chronic diseases is highly specific.^10^ Given the National GI Survey II’s focus on assessing IBS and GI symptom prevalence in the general population, it did not collect data regarding the presence of cirrhosis complications (eg, ascites, hepatic encephalopathy) to minimize question burden for participants. Along similar lines, medication data were not systematically collected, which could impact GI symptomatology. Nevertheless, a key strength of our study is validated assessments of abdominal pain and IBS in a large contemporary sample. Future studies should further explore the effect of cirrhosis severity, specific complications, and cirrhosis-related medications (eg, lactulose) on GI symptoms, helping to identify which patients with cirrhosis are most likely to have these symptoms and how to safely and effectively manage them.

## Supplementary Materials

Supplementary Materials

Extended PDF
