# Supplementary material for: Characterizing Abdominal Pain and Irritable Bowel Syndrome Among Individuals With Cirrhosis: Results of a Nationwide Survey
Source: Gastro Hep Adv. 2026 Apr 20;5(7):100976. doi: 10.1016/j.gastha.2026.100976 (PMC13202564; doi:10.1016/j.gastha.2026.100976)
Supplement: Supplementary Materials [file mmc1.pdf]

Supplementary materials

Participants who met Rome IV criteria but also reported an organic or structural GI condition (i.e., celiac disease, Crohn's disease, diverticulitis, or ulcerative colitis) were excluded from IBS diagnosis, although they remained in the overall study cohort. The GI symptoms included in the survey were chosen based on the NIH PROMIS scales and included abdominal pain, dysphagia, nausea or vomiting, fecal incontinence, bloating, heartburn or acid reflux, diarrhea, and constipation. The PROMIS measures are scored on a T-score metric with a mean of 50 representing the U.S. population and a standard deviation of 10, with higher scores indicating higher severity of symptoms.

Quotas were implemented for age, sex, and region of the US to support recruitment of a study cohort that mirrored the demographics of the US population in 2020; see supplementary material in our prior publication that directly compares the demographics of the study cohort to the US population.[5] The survey was administered by Cint, a research firm that works with various research panels across the US, sampling from almost 20 million panelists who opted to receive survey invitations to participate. Cint has been extensively utilized by researchers globally for a variety of studies, including GI research funded by the Rome Foundation and the National Institutes of Health. Further details on Cint's platform and participant incentives are provided in the supplementary material of our prior publication.[5] Individuals were excluded if they did not complete the survey, had implausible combinations or submitted duplicate entries.

Supplementary Table 1. Demographic and clinical characteristics among adults with and without cirrhosis

Variable	No cirrhosis (n=86,773)	Cirrhosis (n=1,834)	p-value
Age, years			
18-29	22,636 (26.1)	726 (39.6)	
30-39	22,109 (25.5)	473 (25.8)	
40-49	16,340 (18.8)	337 (18.4)	
50-59	14,199 (16.4)	183 (10.0)	
>60	11,489 (13.2)	115 (6.3)	<0.001
Gender			
Male	41,423 (47.7)	753 (41.1)	
Female	43,794 (50.5)	1,021 (55.7)	
Prefer not to answer	1,556 (1.8)	60 (3.3)	<0.001
Race/ethnicity			
Non-Hispanic White	52,461 (60.5)	664 (36.2)	
Non-Hispanic Black	8,876 (10.2)	221 (12.1)	
Hispanic	12,454 (14.4)	412 (22.5)	

Asian	4,685 (5.4)	92 (5.0)	
Other/unknown	8,297 (9.6)	445 (24.3)	<0.001
Education level:			
High school or less	26,016 (30.0)	734 (40.0)	
Some college	21,300 (24.5)	400 (21.8)	
College graduate	27,701 (31.9)	479 (26.1)	
Graduate degree	11,756 (13.5)	221 (12.1)	<0.001
Married	37,109 (42.8)	579 (31.6)	<0.001
Employed or full-time student	54,177 (62.4)	853 (46.5)	<0.001
Total household income:			
$0 to 50,000	37,628 (43.4)	859 (46.8)	
$50,001 to 100,000	24,437 (28.2)	488 (26.6)	
$100,001 to 200,000	12,786 (14.7)	276 (15.0)	
≥$200,001	5,092 (5.9)	145 (7.9)	
Prefer not to say	6,830 (7.9)	66 (3.6)	<0.001
Inflammatory bowel disease	2,360 (2.7)	291 (15.9)	<0.001
Diabetes	7,882 (9.1)	276 (15.0)	<0.001

Fibromyalgia	2,973 (3.4)	147 (8.0)	<0.001
Pancreatitis	1,024 (1.2)	119 (6.5)	<0.001
Alcohol use			
None	46,653 (53.8)	631 (34.4)	
Some days	36,024 (41.5)	1,070 (58.3)	
Every day	4,096 (4.7)	133 (7.3)	<0.001
Tobacco use			
Not at all	63,233 (72.9)	665 (36.3)	
Some days	7,397 (8.5)	703 (38.3)	
Every day	16,143 (18.6)	466 (25.4)	<0.001
